# How does bilingual experience influence novel word learning? Evidence from comparing L1-L3 and L2-L3 cognate status

**DOI:** 10.3389/fpsyg.2022.1003199

**Published:** 2022-11-24

**Authors:** Heng Xue, Renhua Deng, Yanyan Chen, Wenxin Zheng

**Affiliations:** ^1^College of Education, Hebei Normal University, Shijiazhuang, China; ^2^School of Foreign Languages, South China University of Technology, Guangzhou, China; ^3^Sino-Danish College, University of Chinese Academy of Sciences, Beijing, China

**Keywords:** bilingual experience, cross-linguistic similarity, facilitation, interference, L1-L3 cognate status, L2-L3 cognate status

## Abstract

Bilingual experience exerts a complex influence on novel word learning, including the direct effects of transferable prior knowledge and learning skill. However, the facilitation and interference mechanism of such influence has largely been tangled by the similarity of the previously learned word knowledge. The present study compared Chinese-English bilinguals’ paired-associate learning of nonwords in logographic and alphabetic writing systems. The logographic nonwords resemble the form and meaning of L1 Chinese words in varying degrees, being cognates, false cognates, or non-cognates of Chinese. The alphabetic nonwords resemble the form and meaning of L2 English words, being cognates, false cognates, or non-cognates of English. The learning sequence of logographic and alphabetic words was cross-balanced. The learning results were measured in production and recognition tasks. As for learning the logographic nonwords, both the recognition and production results showed that cognates were learned significantly faster than the non-cognates, and the false cognates were also learned significantly faster than the non-cognates. This suggests stronger facilitation rather than interference from L1 on novel word learning. As for learning the alphabetic nonwords, both the recognition and production results revealed that cognates were learned significantly faster than the non-cognates, but false cognates showed no advantage over the non-cognates. This indicates that interference from L2 is stronger than that from L1. Taken together, the results provide new evidence for the dissociable facilitation and interference effects of bilingual experience. These results carry potential educational implications in that learning novel words depends on substantial bilingual experience.

## Introduction

Bilingual experience is one of the main factors that makes word learning different between bilinguals and monolinguals (Del Pilar Agustín-Llach, 2019; Liu et al., 2020; Hirosh and Degani, 2021). When learning a novel word, bilingual experience mainly refers to bilinguals’ extensive experience of mapping novel word form to known concept based on the prior knowledge of the first language (L1) and the second language (L2) as well as their accumulative learning skill of form-meaning mapping (Bartolotti and Marian, 2017; Hirosh and Degani, 2018). Though bilinguals have been found to outperform their monolingual counterparts in vocabulary learning, the bilingual experience of L1 and L2 can exert facilitation or interference effects quite differently (Kaushanskaya et al., 2012; Bartolotti and Marian, 2017). However, the existing research on foreign vocabulary learning is mainly based on the alphabetic writing system (e.g., Bartolotti and Marian, 2019; Zhang et al., 2019; Otwinowska et al., 2020; Vanlangendonck et al., 2020). There are few empirical studies on logographic writing system and even less on both logographic and alphabetic writing systems (though discussed by Ruan et al., 2017; Mok et al., 2018; Liu et al., 2018; Richlan, 2020). Thus, the facilitation or interference effect of bilingual experience may be tangled by the similarity of writing systems (Eng et al., 2019; Jiang, 2021). In this study, 41 Chinese-English bilinguals participated in our experiment to learn logographic and alphabetic novel words through paired-associate learning and completed recognition and production tasks to measure the learning outcomes (Marecka et al., 2021). The logographic nonwords share different degrees of form and meaning overlapping with L1 Chinese, such as “焝纱.” It refers to “wedding dress” in English and slightly differs in radicals from the original L1 Chinese word “婚纱.” The alphabetic nonwords share different degrees of form and meaning overlapping with L2 English, such as “pandda.” It refers to “panda” in English but slightly differs in the spelling of the original L2 word “panda.” This study is unique as the influence from L1 and L2 can be disentangled from learning alphabetic or logographic novel words based on a within-subject experiment design, contributing to identifying the facilitative and interferent mechanism of bilingual experience on novel word learning.

### Learning novel words of different cognate status

Learning a new word in a foreign language means acquiring knowledge of the word form and mapping the form to the concept (Schmitt, 2019; Nation, 2020). To measure the learning outcome of such knowledge, both recognition and productive aspects are assessed. The recognition task is used to access whether the learner can recognize the form-meaning mapping of a word, whereas the production task evaluates the learner’s ability to produce the word form. Existing studies have basically confirmed that specificity, frequency, and word presentation have an impact on novel word learning (Wang et al., 2020). Thus, it is necessary to use nonwords or artificial words, controlling their semantic specificity, logographic, and other essential information to study the learning effect.

Words to be learned in a foreign language may share varying degrees of overlap with the previously-learned words and thus can be classified into three types of cognate status, i.e., cognate, false cognate, and non-cognate (Simpson Baird et al., 2016; García et al., 2020). A cognate is a word whose form and meaning are almost the same in two different languages, such as the Chinese word “小说” and the Japanese word “小説” (both refer to the meaning of “fiction”) as well as the English word “actor” and the French word “acteur” (both refer to the meaning of “actor”). False cognates refer to two words in different languages that have quite similar forms but have different meanings, such as the word “大手” in Chinese and Japanese (refers to “big hands” in Chinese and “large enterprises” in Japanese) as well as the word “magazine” in English and “magasin” in French (it refers to “magazine” in English and “shop” in French). Non-cognates are words that do not share a significant formal similarity with L1 or L2 words. Thus, the cognates are well-matched with the prior language experience and the false cognates are the mismatched ones, when non-cognates are used as baselines for comparison (Iniesta et al., 2021; Marecka et al., 2021).

Cognate status is a well-explored topic in foreign language learning (Van Hell and De Groot, 1998; Hirosh and Degani, 2018). Studies have used non-identical spellings to replace identical spellings in experiments to identify the word form learning more precisely (Dijkstra et al., 2010; Arana et al., 2022). Cognates show advantages over non-cognates in recognition and production tasks in most studies (e.g., Lemhöfer et al., 2004; Van Hell and De Groot, 2008). Recently, the cognate facilitation effect has been reported to be moderated by the bilingual experience (Iniesta et al., 2021). Cross-language orthographic errors have been observed as evidence of cognate interference (Muscalu and Smiley, 2018). Besides, false cognates have also been used to clarify the influence of the previously-learned form and form-meaning mapping in bilingual experience (Marecka et al., 2021; Elias and Degani, 2022). Bilingual experience brings in the transferable knowledge and representations, and also the abilities to acquire the knowledge and create the representations (Hirosh and Degani, 2021; Marecka et al., 2021). Nevertheless, the form-meaning mismatch of false cognates inevitably costs extra efforts to differentiate and re-match the form-meaning mapping (Janke and Kolokante, 2015). Notably, the facilitation and interference from a previously learned language can be interwoven, competing to assist or hinder the novel word learning outcome. Therefore, learning L1-L3 and L2-L3 false cognates, respectively, can help explicate the subtle facilitation-and-interference mechanism of the bilingual experience.

### Learning novel words with bilingual experience

Bilingual experience is formed by the accumulation of knowledge, acquisition, and regular use of two languages (Kroll et al., 2014; Subramaniapillai et al., 2019). Hirosh and Degani (2018) proposed a direct–indirect framework to clarify the effects of bilingual experience on learning novel languages. Direct effects include firstly those transferable knowledge and representations from known languages, and secondly the abilities to acquire the knowledge and create the representations. Indirect effects refer to the additional mediating role of bilingual experience as an advantage in a broader sense, such as cognitive and social abilities. Learning novel words in additional languages involves both the direct and indirect effects of bilingual experience. The direct effects critically depend on the degree of cross-linguistic similarity, i.e., the more similar the more direct effects (Antoniou et al., 2015). Besides, the direct effects also depend on the status of the two previously learned languages, i.e., the more frequently a particular pattern of mappings is experienced, the stronger learning advantage can be expected (Koda and Miller, 2018). Nevertheless, the indirect effects mainly refer to the bilinguals’ learning advantage over their monolingual counterparts as well as the developmental changes of multilingual language learners. Therefore, in order to reveal the facilitation and interference of the known languages on the to-be-learned language, the direct effects should be the focus of research.

Studies so far provided little conclusive evidence on how the bilingual experience facilitates or interferes with subsequent word learning. Research has shown both L1 and L2 benefit further word learning. Bartolotti and Marian (2017) found that novel word learning benefited from both L1-English and L2-German, in which participants used an English keyword for Englishlike words and a German keyword for Germanlike words. Besides, a novel word’s similarity to both L1 and L2 did not provide an additional learning benefit. The direct effects of bilingual experience may even be more complex. Mulík and Carrasco-Ortiz (2021) compared the phonological activation of L2 cognates and L1 cognates through event-related potentials (ERPs). Their research found that both L1 Spanish and L2 English facilitated learning novel L3 Slovak words in similar behavioral results but with different electrophysiological results. Evidence for a stronger facilitative role of L1 originated mainly from translation-related research. Hirosh and Degani (2021) found that bilinguals learned novel words better through L1 translations rather than L2 translations. Bogulski et al. (2019) also found that bilingual advantage in vocabulary learning depended on learning *via* the L1 or dominant language because learning *via* the L1 allows bilinguals to engage regulatory skills that benefit further vocabulary learning. Another line of research concerned the interferent effect of the bilingual experience. They found the L2 (status) effect rooted in the model of inhibitory control (Green, 1986, 1998; de Bot and Jaensch, 2015), which predicted the inhibition of the highly proficient language (usually L1) to retrieve the less developed languages (L2 and L3), thus leaving L2 and L3 in a competing condition with L1.

Furthermore, the role of bilingual experience can be tangled by the similarity among the languages learned and to be learned. Extensive studies have investigated monolinguals or bilinguals using alphabetic writing systems as L1 and even L2 experience, but more attention has been recently paid to those using logographic writing systems (see Table 1 for the relevant articles retrieved from Web of Science since 2017). Hsieh et al. (2017) found that although Japanese-Chinese bilinguals have a bilingual automatic activation mechanism similar to that of alphabetic bilinguals, the process of logographic recognition requires more neural mechanisms for semantic selection and suppression between cognates and false cognates. Zhang et al. (2021) found that Chinese-Japanese speakers’ cognate awareness systematically predicts the vocabulary learning outcomes of Japanese words. Nevertheless, there is still a lack of research on how orthographically different L1 and L2, respectively, affect the learning outcomes of novel words similar to either L1 or L2. It would be more transparent to probe into the role of bilingual experience with participants of different writing systems.

**Table 1:** Major relevant articles concerning form-meaning mapping since 2017.

Author(s)	Publication year	Language experience			Writing system	Results	
		L1	L2	L3		Recognition	Production
Otwinowska and Szewczyk	2019	Polish	English		Same	Translation: C > NC > FC	
Otwinowska et al.	2020	Polish	English		Same	Cognate awareness did not boost learning cognates.	
Marecka et al.	2021	Polish	Nonword		Same	C > FC ≈ NC	C > FC > NC
Iniesta et al.	2021	Spanish	English		Same	Word dictation task English: C < NC; Spanish: C > NC
	Li and Golla	2021	Spanish	English		Same	Naming: C > NC
	Robinson Anthony et al.	2022	Spanish	English		Same	Language dominance was found to predict crosslinguistic (cognate) facilitation from Spanish to English.
	Muylle et al.	2022	Dutch	English		Same	
C > NC	Allen	2019	Japanese	English		Different	Cognate frequency effect was found.
	Zhang et al.	2019	Chinese	English		Different	C > NC
	Bartolotti & Marian	2017	English	German	Nonword	Same	C > NC
C > NC	Cenoz et al.	2021	Basque	Spanish	English	Same	Cognate awareness did not boost learning cognates.
	Hirosh and Degani	2021	Hebrew	English	German	Different	With L2 translation (Error rates): C > FC > NC; with L1 translation (Error rates): C > NC ≈ FC; with L1, L2 translation (RTs): C > FC ≈ NC	With L1, L2 translation (Error rates, RTs): C > NC ≈ FC;

C *=* cognate; FC = false cognate; NC = non-cognate; “>” means the performance is better.

### The current study

**Figure 1:**
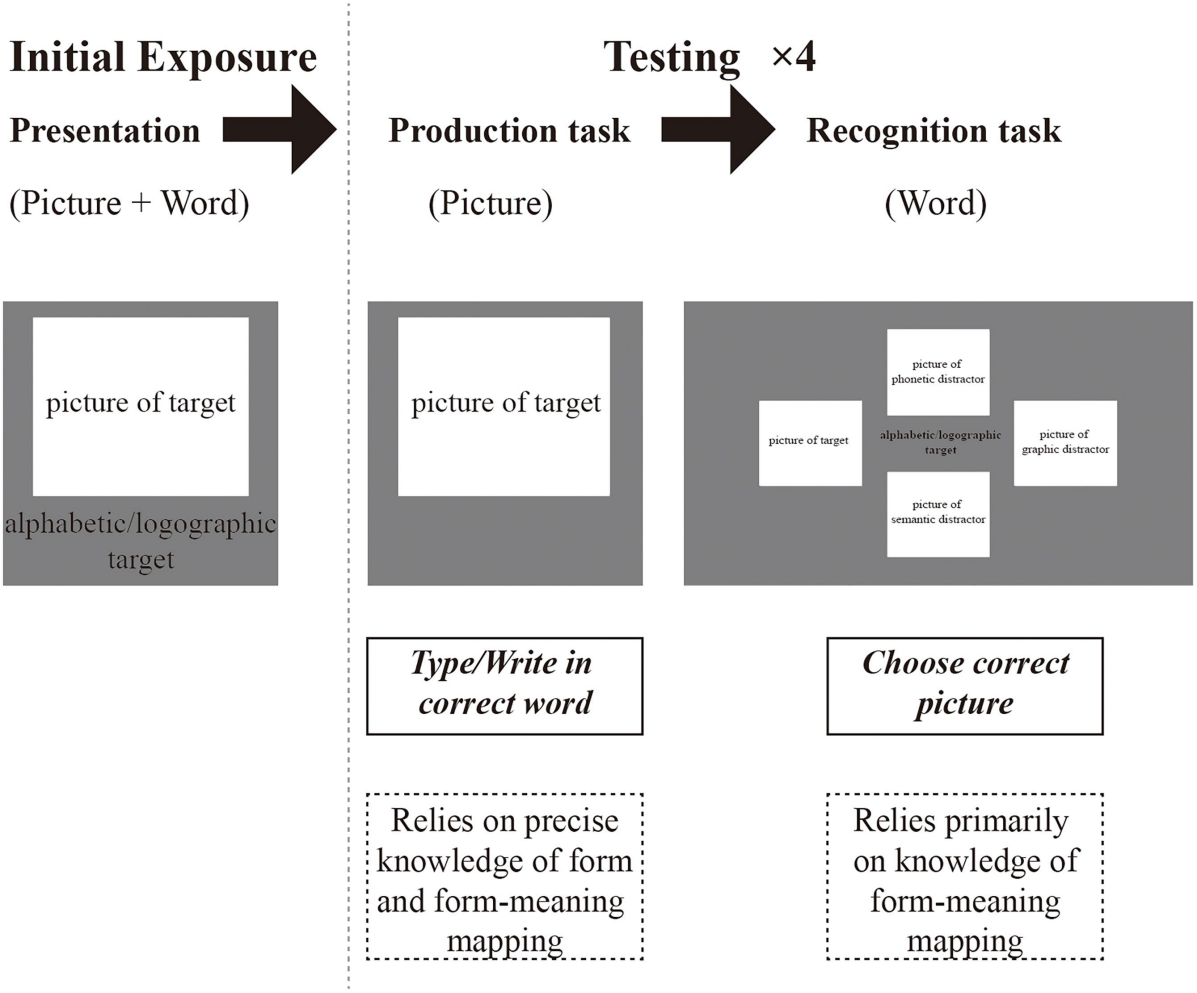
A schematic overview of the paradigm design.

The current study explored the role of prior bilingual experience in learning L3 alphabetic and logographic novel words. To this end, we used a word-learning experiment in which participants were continuously visually exposed to, tested on, and provided feedback about the forms and meanings of the target words (Marecka et al., 2021). Furthermore, we used artificially created logographic and alphabetic nonwords to disentangle L1-L3 cognate status and L2-L3 cognate status. The learning process of alphabetic and logographic words was investigated through cross-balancing within-subject design. Chinese-English bilingual participants learned the nonwords through paired pictures of objects which represented the novel words’ meanings. The novel words included cognates, false cognates, and non-cognates of the learners’ L1 Chinese and L2 English, respectively. It has been reported that at least 6 to 16 encounters with a word are needed to learn it (Nation, 2013). In our pilot study, the participants showed a decline in attention after 10 times of learning. Thus, our experiment paradigm exposed learners to each new word 9 times. The alphabetic and logographic learning blocks were the same. All the participants’ learning sequences of alphabetic and logographic blocks were balanced. At the onset of the study, participants were presented with an exercise block to familiarize them with the production and recognition tasks. Then participants were presented with each target picture-word pair, one at a time. After this initial offline presentation, the tasks started, and the participants performed a series of production blocks interleaved with recognition blocks (see Figure 1 for a schematic overview of the paradigm design). In the recognition task, together with the target picture, three distractors were also presented, i.e., a semantic distractor, a graphic distractor, and a phonetic distractor. After each trial, the participants were given feedback on the accuracy of their response, so the production and recognition blocks both tested and trained the participants. The accuracy of the last round of the production task and the reaction times (RTs) of the correct answers in the last round of the recognition task was used for statistical analysis. One novel aspect of our study is that we combined testing of the alphabetic and logographic writing systems with the same participants, which has rarely been done in experiments so far.

## Materials and methods

### Experiment design

This experiment is based on the experiment designed by Marecka et al. (2021), which examined the Polish natives learning alphabetic nonwords. In the current experiment, Chinese-English bilinguals learned both the alphabetic and logographic new words. PsychoPy 3 (Peirce et al., 2019) was used to present the materials on a computer screen, a computer keyboard was used to collect the RTs in recognition tasks, and Han Wang electronic writing tablet served to record the results in production tasks. In this study, the dependent variables were the test scores of the results, i.e., accuracy in production tasks as well as RTs in recognition tasks. The independent variables were word types (cognate, false cognate, and non-cognate) and writing systems (alphabetic and logographic).

The basic assumption of the research is that learners’ prior language experience may influence L3 vocabulary learning differently. The learning outcomes of different word types should be compared within the logographic or the alphabetic division to reveal the language experience of L1 and L2. If L1-L3 cognates and L2-L3 cognates are learned faster than L1-L3 and L2-L3 non-cognates respectively, the bilingual experience can be proved to exert facilitative cross-linguistic influence independently. If L1-L3 false cognates and L2-L3 false cognates are learned faster than L1-L3 and L2-L3 non-cognates respectively, the bilingual experience can be proved to exert more facilitative rather than interferent cross-linguistic influence independently. If the patterns of learning outcomes differ between the writing systems, the bilingual experience can be proved to act in different modes.

### Participants

Forty-one Chinese-English bilinguals aged from 18 to 25 were recruited for this study. All participants have been learning English at school since the age of 9 to 11. All participants did LexTALE (Lemhöfer and Broersma, 2012), a test of vocabulary knowledge for speakers of English as a second or foreign language. The results of LexTALE ranged from 43.75 to 87.5%, indicating that participants’ proficiency ranged across three levels, i.e., upper advanced, upper intermediate, and lower intermediate (Lemhöfer and Broersma, 2012). They also finished a bilingual language use profile (Gertken et al., 2014) to research their everyday language use of alphabetic and logographic words. According to the language use profile results, all participants used Chinese as the dominant language.

### Materials

#### Creating alphabetic stimuli

The target alphabetic stimuli were 24 nouns paired with pictures (6 cognates, 6 false cognates, and 12 non-cognates of L2 English as shown in Supplementary material). Based on the 2000 common English nouns in the Chinese education curriculum, this study selected nouns of 5 to 7 letters in length. By replacing, adding, or subtracting one letter, the alphabetic stimuli were words of 6 letters in length (Bartolotti and Marian, 2019). For example, “banana” can be changed into “benana.” Firstly, approximately 300 English nouns were selected. Next, the words’ concreteness and imageability were rated *via* a 5-point Likert scale (5 indicates the most concrete or imaginable) by a group of 8 Chinese-English bilinguals who would not participate in the following experiment. The concreteness or imageability ratings lower than 4 were eliminated. Then the words were put into CLEARPOND to search their frequency and neighbor size (Marian et al., 2012). Those frequencies over 25 occurrences per million and neighbor sizes over 4 were eliminated. Finally, their Chinese translations were matched, and their translation frequencies were log-transformed and controlled between 3.75 to 4.25 per million words in BLCU Chinese Corpus^1^, and their translations were all two-character Chinese words. Only 30 nouns were reserved as the alternative meaning of the 24 nonwords in the experiment.

From the selected words, the 6 cognates and 6 false cognates were randomly assigned. The 6 cognates were matched to their original meaning in English. For example, “pandda” was assigned as cognates, meaning “panda.” The meanings of the 6 false cognates were randomly selected among the 30 mostly concrete and imaginable nouns rated previously. False cognates’ meanings differed remarkably from their initial meanings.

A hundred nonwords with 6 letters were first generated and their neighbor sizes were controlled to less than 4 in the ARC nonword database (Rastle et al., 2002). The non-cognates were 6 non-wordlike non-cognates and 6 wordlike non-cognates based on whether the form is similar to an English word. Finally, the meaning of the words selected before (30 nouns) was randomly assigned to the words generated in ARC.

#### Creating logographic stimuli

Similar to the alphabetic stimuli, the target logographic stimuli were 24 nouns paired with pictures (6 cognates, 6 false cognates, and 12 non-cognates of L1 Chinese as shown in Supplementary material). Based on the common Chinese nouns in the Chinese education curriculum, this study only used the two-characters nouns as the meaning of the 24 nonwords. Firstly, those frequencies were log-transformed and controlled between 3.75 to 4.25 per million words in BLCU Chinese Corpus. Next, the words’ concreteness and imageability were rated *via* a 5-point Likert scale (5 indicates the most concrete or imaginable) by a group of 8 Chinese-English bilinguals who would not participate in the following experiment. The rating of imageability and concreteness less than 4 were deleted.

From the selected words, the meaning of 6 cognates, 6 false cognates, and 12 non-cognates was randomly assigned. Non-characters were created by randomly combining the phonetic and semantic radicals of the actual character stimuli following orthographic rules (Yum et al., 2014). Six cognates were created at first. For example, the form of the cognate “焝纱,” which is the transformation of the “婚纱,” was created by replacing the radical “女” into “火.” The form of the six false cognates was the same as the cognates. Differently, false cognates’ meanings differed remarkably from their initial meanings. For example, the false cognate “烧烤” means “梨子” in the experiment, which is transformed from “烧烤” by replacing “火” into “女.” Twelve non-cognates were divided into 6 wordlike non-cognates and 6 non-wordlike non-cognates. The wordlike non-cognates were transformed from a real Chinese word by changing two or three radicals, while the non-wordlike non-cognates did not follow the structure of Chinese words. The orthographic neighborhood size of 18 nonwords (6 cognates, 6 false cognates, and 6 wordlike non-cognates) was controlled between 20 and 30 (Dong et al., 2015). In addition, all the nonwords’ strokes were controlled between 11 and 26. Finally, Truetype, a special character editing program in Windows 10, was used to present the nonwords in picture format.

#### Selecting associative pictures

Pictures were selected to indicate the meanings of the 48 logographic and alphabetic stimuli. Another 130 pictures were selected for the distractors in the recognition task. Pictures for the logographic and alphabetic stimuli were used both in the learning session and the recognition task in the test session. Pictures for the distractors were used only in the recognition task. All the pictures were from Cambridge online dictionary^2^ and Bing picture database^3^. The pictures were piloted through an online translation task by 12 Chinese-English bilinguals who would not participate in the formal experiment. Thus, the pictures were validated that they were not ambiguous.

### Procedure

Participants took part in a computerized word-learning task. They were asked to learn 24 alphabetic nonwords and 24 logographic nonwords, respectively. The interval between the alphabetic experiment and the logographic experiment was 1 day. The sequence of the alphabetic experiment and logographic experiment was balanced. The procedure could be divided into 4 parts, i.e., exercise trials, initial presentation block, production block, and recognition block. The sequence of the alphabetic and logographic experiments was balanced, and each participant’s interval of the logographic and alphabetic experiment was 1 day.

The first block was the exercise trials. Five pictures were displayed in the center of the computer one after another. Participants were asked to write the correct word for the picture. It is similar to a production task. Then 5 words were displayed randomly in the center of the computer. Participants were asked to choose the correct picture using the keyboard. It is similar to the recognition task. All 5 words were irrelated and were actual words paired with pictures.

The second block was the initial presentation block. All 24 nonwords were displayed on the screen randomly with the pictures they represented. Before the presentation, the instruction had told participants to memorize the nonwords. Participants can press “space” to cut the instruction. Next, the screen presented with fixation “+” for 500 ms to remind participants to pay attention. Then on the center of the screen presented a picture with a nonword below it for 1,500 ms.

After the initial presentation block was the production block. All 24 nonwords were displayed on the screen randomly with the pictures. However, there was no time limit for it. Participants were told to write the answer (nonword) as much as they could or leave them blank if they could recall nothing. There was also a fixation pattern “+” for 500 ms before every picture to remind them. If the answer was correct, the feedback would present “Correct” for 500 ms, followed by the correct nonword and the corresponding picture for 500 ms. If the answer was wrong, the feedback would present “Sorry, you are wrong.” for 500 ms, followed by the correct nonword and corresponding to the picture for 500 ms. It is worth noting that only the alphabetic production task had the feedback but not in the logographic production task because the computer could not recognize the handwriting of logographic nonwords.

**Figure 2:**
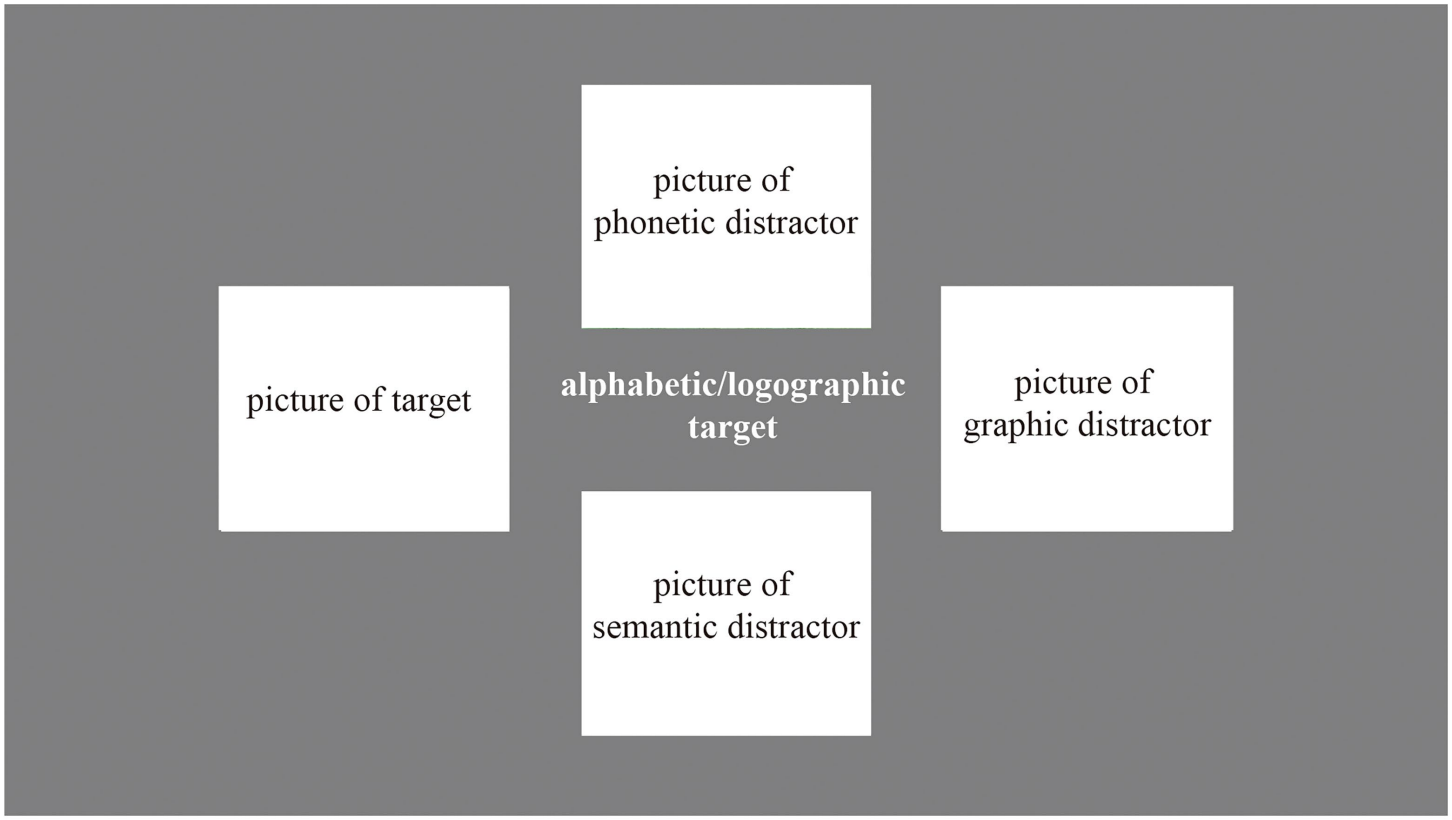
The target, semantic distractor, graphic distractor, and phonetic distractor for the alphabetic and logographic nonwords.

Next, was the recognition block. In the recognition block, participants were told to choose the corresponding picture to which the nonword refers. There were 4 kinds of pictures (see Figure 2), i.e., target (the correct item), phonetic distractor (the picture corresponding to the word which pronounces similarly to the nonword), semantic distractor (the picture corresponding to the word whose meaning is similar to the nonword), and graphic distractor (the picture corresponding the word which looks like to the nonword). They were randomly displayed on the nonword’s top, left, down, and right. We counterbalanced the position of the four categories of pictures across trials and blocks. The participants were asked to press the arrow key to choose the picture. Each nonword was presented for 4,000 ms, meaning that participants must choose the answer in 4,000 ms or it would be regarded as wrong. After that was feedback similar to the production block, which included “Correct” or “Sorry, you are wrong.” for 500 ms and the nonword with the correct picture for 1,500 ms. All 24 nonwords were in a random sequence.

The recognition and production test blocks were interleaved. There were 4 loops, which means 4 production blocks and 4 recognition blocks. Participants learned the nonwords 9 times in total (1 time in the initial presentation, 4 times in the production block, and 4 times in the recognition block).

The authors of this article looked through every incorrect response. Participants were interviewed after the experiments to make sure of their performance. The questions asked are presented below:

1. In the recognition task, why did you choose this incorrect answer?
2. In the recognition task, was there any difference in learning the alphabetic and logographic words? And why?
3. In the production task, what made you write the word in this incorrect way?
4. In the production task, was there any difference in learning the alphabetic and logographic words? And why?

### Data analysis

#### Production data analysis (accuracy)

In the alphabetic production test blocks, this study calculated the normalized Levenshtein Distance (nLD) between the correct nonword and the nonword typed by the participants. The Levenshtein Distance (LD) is a metric that indexes the total number of insertions, deletions, and substitutions necessary to transform one string of letters onto another (Levenshtein, 1966). For example, the LD between the word “apple” and “epple” is 1 because they differ in one letter. The LD between the word “apple” and “abble” is 2 because they differ in two letters. The nLD is the LD between the two words divided by the number of target word letters. For example, the nLD between the word “apple” (as the target) and “aple” is 1/5. This study calculated the production score using nLD.

In the alphabetic production test blocks, the score of every nonword was the average of 4 production tasks’ scores. Every production score could be calculated by nLD (production score = 1 − nLD). For example, the nLD between the word “apple” and “epple” is 1/5, and its production score is 4/5. The nLD between the word “apple” and “abble” is 2/5, and its production score is 3/5. If a participant wrote “abbla,” “abble,” “appla,” “apple,” his or her score of the word “apple” should be 0.7.

The scoring of the logographic production test was performed similarly but adjusted to the features of logographic words. Logographic words were firstly separated into characters, and radicals of interweaved strokes formed each character. Thus, According to Barcroft (2002), the scoring method was adopted to consider partial correctness, i.e., the radicals. The partly reproduced words were given 0.25, 0.5, or 0.75 based on the radicals. The produced words were scored as 1. For instance, the Chinese character “婚” could be divided into two radicals, i.e., “女” and “昏.” A two-character nonword “焝纱” could be considered to disassemble into four parts, i.e., “火,” “昏,” “纟,” and “少.” If the target word was wrongly written as “婚纱” in the first production task and correctly written as it should be in the remaining production tasks, the score would be 3/4 in the first production task and 1 in each remaining production task. The final score of this word production would be 0.94 (3.75/4).

#### Recognition data analysis (reaction time)

In the recognition test, both alphabetic and logographic blocks measured the accuracy of response and its Reaction Times (RTs) as the score of the recognition task. The RTs of every nonword were log-transformed. To balance the speed and accuracy, we only analyzed the correct-chosen nonwords. After calculating all the words’ scores, the average score of cognates, false cognates, and non-cognates were calculated.

## Results

All relevant data, as well as analysis scripts, are available on the OSF platform.^4^

### Production blocks

Mixed model analyses were conducted on R software (version 4.1.2; R Core Team, 2021), using lmer functions from the lme4 package (Bates et al., 2015). The models included dummy-coded fixed effects of writing system as a within-subject variable (alphabetic vs. logographic, with alphabetic set as the reference), word type also as a within-subject variable (cognate, false cognate, non-cognate, with the non-cognate set as the reference), and the interaction between writing system and word type. The formula of the maximal model was lmer[score.pro ~ word type * writing system + (1 + word type + writing system| Subject) + (1| Item)]. The word type and writing system were not set as random slopes for item because an item was presented in one writing system and one word type. The word type and writing system were set as random slopes for subject because a subject responded to two writing systems and three types of words. The maximal model was fitted using the buildmer function in the buildmer package (Version 1.3; Voeten, 2019) in R, which uses the lmer function from the lme4 package (Bates et al., 2015). Using backwards stepwise elimination, the buildmer function starts from the most complex model and systematically simplifies the random structure until the model converges. This resulted in a random intercept for word and subject and a random slope for word type and writing system over subject. The fixed part consisted of the writing system (alphabetic vs. logographic), word type (non-cognate, cognate, false cognate), and their interaction. Model formula is: lmer[score.pro ~ word type * writing system + (1| Item) + (1 + word type + writing system| Subject)]. Model intercept reflects the score of the alphabetic non-cognates. The model for the production block is presented in Table 2 (fixed effects) and Table 3 (random effects). The outcome variable in the model is the score for each item. In general, the ANOVA shows the production task significantly differed between logographic and alphabetic nonwords (*F* = 261.58, *p* < 0.001), indicating that L1-L3 cognate status plays a different role from L2-L3 cognate status. A simple effect (see Table 4) is tested showing that cognates were learned significantly faster than non-cognate in both the alphabetic block (estimate: 0.40, *SE* = 0.05, *t* = 7.80, *p* < 0.001) and logographic block (estimate: 0.63, *SE* = 0.05, *t* = 12.03, *p* < 0.001). The major difference between learning alphabetic and logographic nonwords lies in L1-L3 and L2-L3 false cognates. As Table 4 shows, logographic false cognates showed difference from non-cognates (estimate: 0.34, *SE* = 0.05, *t* = 6.69, *p* < 0.001) while alphabetic did not differ (estimate: 0.08, *SE* = 0.05, *t* = 1.53, *p* = 0.14).

**Table 2:** Fixed effects from linear mixed model of score with writing systems and word type as fixed effects in the production tasks.

Effect	Estimate	*SE*	*df*	*t*	*p*
*(Intercept)*	0.49	0.04	74.10	13.24	**< 0.001** ^***^
Logographic vs. alphabetic	-0.30	0.04	54.99	−6.72	**< 0.001** ^***^
False cognate vs. non-cognate	0.08	0.05	44.26	1.52	0.13
Cognate vs. non-cognate	0.40	0.05	47.75	7.78	**< 0.001** ^***^
Logographic vs. alphabetic * false cognate vs. non-cognate	0.26	0.07	42.05	3.74	**< 0.001** ^***^
Logographic vs. alphabetic * cognate vs. non-cognate	0.22	0.07	42.06	3.17	**0.002** ^**^

**p < 0.01 and ***p < 0.001.

**Table 3:** Random effects from linear mixed model of score with item and subject as random effects in the production tasks.

Groups	Name	SD	Variance	ICC
Item	*(Intercept)*	0.10	0.01	0.00
Subject	*(Intercept)*	0.15	0.02	0.31
	Writing system (logographic)	0.11	0.01	
	Word type (false cognate)	0.06	0.00	
	Word type (cognate)	0.09	0.01	
Residual		0.18	0.03	

**Table 4:** Simple effects in the production tasks.

Writing system	Contrast	Estimate	*SE*	*df*	*t*	*p*
Alphabetic	False cognate vs. non-cognate	0.08	0.05	22.30	1.53	0.14
	Cognate vs. non-cognate	0.40	0.05	24.20	7.80	< 0.001^***^
Logographic	False cognate vs. non-cognate	0.34	0.05	22.30	6.69	< 0.001^***^
	Cognate vs. non-cognate	0.63	0.05	24.20	12.03	< 0.001^***^

***p < 0.001.

**Figure 3:**
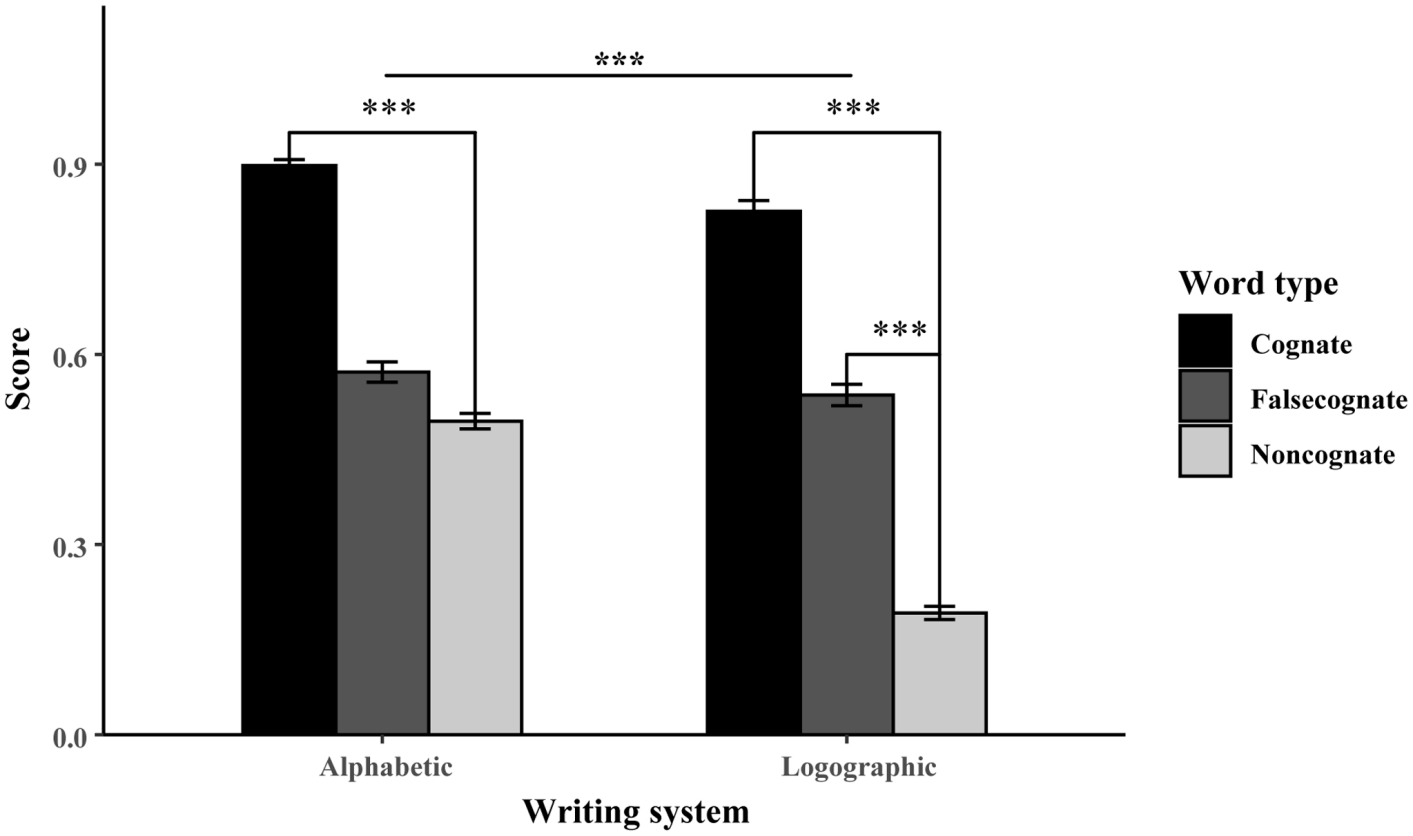
The alphabetic and logographic score for the cognate, false cognate and non-cognate in the production block.

Figure 3 compares the score for the three word types in alphabetic and logographic writing systems. Taken together, the results in production tasks display the significant difference between the two writing systems. Therefore, the results in production task indicate that participants’ bilingual experience facilitated the production of well-matched novel words, i.e., the L1 and L2 cognates. Furthermore, in learning the form-meaning mismatched novel words, interference can be overcome by the facilitation of the participants’ dominant language, i.e., L1 Chinese; thus, the L1-L3 false cognates were learned faster than the non-cognates. However, the facilitation and interference from L2 English were quite balanced in learning L2-L3 false cognates, leaving no significant advantage in the production task.

### Recognition blocks

The formula of the maximum model was lmer[RTs ~ word type * writing system + (1 + word type + writing system| Subject) + (1| Item)]. The model selection process of recognition task was similar to the production task. It turned out the best model was lmer[RTs ~ word type * writing system + (1| Item) + (1 + writing system + word type| Subject)]. Model intercept reflects the RTs (log-transformed) of the alphabetic non-cognates. The RTs for the recognition blocks are tested and log-transformed before analyses to reduce skew in the distribution. The model is presented in Table 5 (fixed effects) and Table 6 (random effects). Overall, the pattern of the results is similar to that of the production task. A further *post–hoc* comparison between cognates and false cognates (estimate: 0.24, *SE* = 0.05, *t* = 5.12, *p* < 0.001) indicates significant differences. A simple effect (Table 7) is tested suggesting that there was no difference between non-cognate and false cognate in alphabetic writing system (estimate: 0.00, *SE* = 0.06, *t* = 0.05, *p* = 0.96) but the significant difference was detected in logographic writing system (estimate: −0.16, *SE* = 0.06, *t* = −2.79, *p* = 0.01). Similar results were also found in the production tasks. As Table 7 shows, cognates were learned faster than non-cognates in both alphabetic (estimate: -0.30, *SE* = 0.06, *t* = −5.17, *p* < 0.001) and logographic writing systems (estimate: −0.34, *SE* = 0.06, *t* = −5.84, *p* < 0.001).

**Table 5:** Fixed effects from linear mixed model of RTs with writing systems and word type as fixed effects in the recognition tasks.

Effect	Estimate	*SE*	*df*	*t*	*p*
*(Intercept)*	0.50	0.04	69.46	12.38	< 0.001^***^
Logographic vs. alphabetic	0.11	0.05	51.93	2.25	0.03^*^
False cognate vs. non-cognate	0.00	0.06	43.14	0.05	0.96
Cognate vs. non-cognate	−0.30	0.06	45.44	−5.17	< 0.001^***^
Logographic vs. alphabetic * false cognate vs. non-cognate	−0.16	0.08	42.00	−2.02	0.05^*^
Logographic vs. alphabetic * cognate vs. non-cognate	−0.04	0.08	42.00	−0.49	0.63

*p < 0.05 and ***p < 0.001.

**Table 6:** Random effects from linear mixed model of RTs with item and subject as random effects in the recognition tasks.

Groups	Name	*SD*	Variance	*ICC*
Item	*(Intercept)*	0.11	0.01	0.25
Subject	*(Intercept)*	0.14	0.02	0.35
	Writing system (logographic)	0.11	0.01	
	Word type (false cognate)	0.05	0.00	
	Word type (cognate)	0.08	0.01	
Residual		0.19	0.04	

**Table 7:** Simple effects in the recognition tasks.

Writing system	Contrast	Estimate	*SE*	*df*	*t*	*p*
Alphabetic	False cognate vs. non-cognate	0.00	0.06	43.10	0.05	0.96
	Cognate vs. non-cognate	−0.30	0.06	45.40	−5.17	< 0.001^***^
Logographic	False cognate vs. non-cognate	−0.16	0.06	43.30	−2.79	0.01^**^
	Cognate vs. non-cognate	−0.34	0.06	45.60	−5.84	< 0.001^***^

**p < 0.01 and ***p < 0.001.

**Figure 4:**
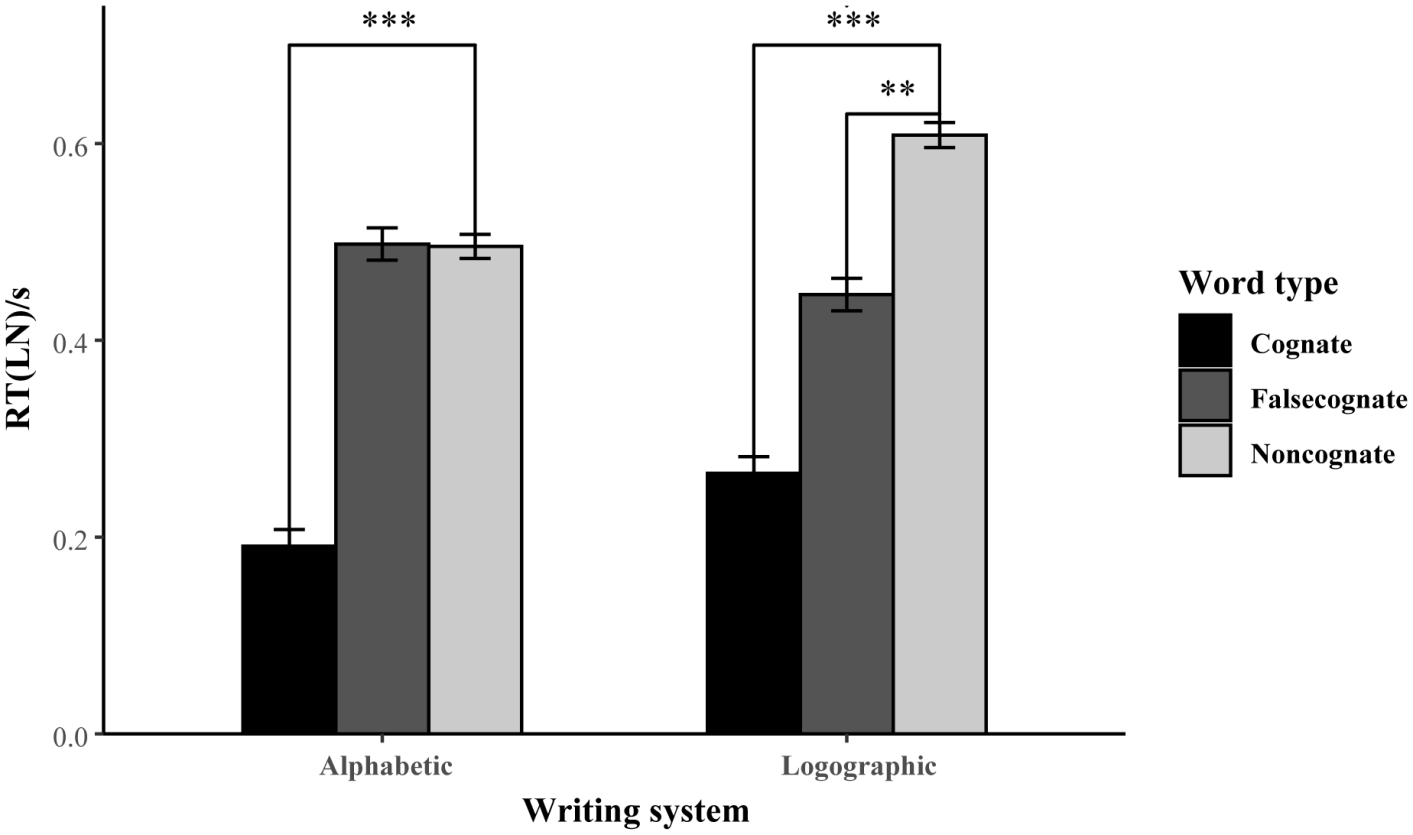
The alphabetic and logographic RTs for the cognate, false cognate and non-cognate in the recognition block.

Figure 4 compares the RTs for the three word types in alphabetic and logographic writing systems. Taken together, the results in recognition tasks display less difference between the two writing systems in learning cognates. Both L1 and L2 facilitated the well-matched novel learning. However, the results of the false cognates are similar to those of the production task, revealing that there was more facilitation than interference from L1, while the facilitation and interference from L2 were quite balanced.

## Discussion

In this study, we examined the facilitative and interferent effects of bilingual experience on novel word learning. To this end, nonwords were created either in the logographic form similar to L1 Chinese or in the alphabetic form like L2 English, which can be further divided into cognates, false cognates, and non-cognates in either L1-L3 or L2-L3 cognate status. An L1-L3 cognate has a slightly different logographic form and completely identical meaning to the original L1 Chinese word. In the same vein, an L2-L3 cognate almost coincides with the alphabetic L2 English words in form and meaning. An L1-L3 false cognate has the logographic form that coincides with the already learned L1 but has a different meaning. Similarly, an L2-L3 false cognate shares the alphabetic form of the original L2 word but not the meaning. The learning outcomes of cognates and false cognates were compared to their non-cognates individually in the logographic block and alphabetic block. Therefore, the learning outcome of the L1-L3 and L2-L3 cognates can be individually traced from either L1 Chinese or L2 English. The learning outcomes were made up of the participants’ production scores and recognition RTs. The production task is designed to test the precise knowledge of form and form-meaning mapping. The recognition task is designed to mainly test the knowledge of form-meaning mapping and a small amount of form knowledge. Such an experimental design functioned to examine the direct effects of bilingual experience on novel word learning, i.e., the transferable knowledge and representations from the previously learned word forms and concepts as well as the skills of learning them (Hirosh and Degani, 2018).

The results show that the direct facilitation effects from L1 and L2 can be separately traced from the logographic and alphabetic novel word learning. Both the L1-L3 and the L2-L3 cognates were learned faster than their non-cognate counterparts in the production and recognition tasks. The results indicate that learners will automatically search for, detect, and use similar information between the languages known and those to be learned. In another word, the bilingual experience can exert its facilitation effect in a dissociable way with the logographic and alphabetic novel words. However, the sophistication of the facilitation and interference from bilingual experience can be more subtly revealed by the learning outcomes of false cognates. In recognition, the learning of false cognates is mostly influenced by the mismatched form-meaning mapping; in production, it’s affected by both the form overlap and the mismatched form-meaning mapping (Janke and Kolokante, 2015; Marecka et al., 2021). The current study found no significant difference between L2-L3 false cognates and L2-L3 non-cognates in both the recognition and production tasks. The interference from L2 mismatched form-meaning mapping was possibly offset by the facilitation of the form overlap. Quite differently, a stronger facilitation effect was observed from L1 Chinese, leading to the result that the L1-L3 false cognates were learned significantly better than the non-cognates in both the recognition and the production tasks. Therefore, the current study contributes to providing new evidence to the facilitation and interference mechanism of how bilingual experience affects novel word learning when L1 and L2 word knowledge is not explicitly activated as translations (Cenoz et al., 2021; Hirosh and Degani, 2021).

### The facilitation effect of bilingual experience in learning cognates

The current study provides new evidence of bilingual experience with orthographically different languages. Consistent with previous studies, a learning advantage for cognates is found both in laboratory settings and classroom settings (e.g., Bartolotti and Marian, 2019; Zhang et al., 2019; Otwinowska et al., 2020; Vanlangendonck et al., 2020). Regardless of the different writing systems of the bilingual experience, cognates were the quickest to be recognized and produced. Such facilitative effects were not moderated by the difference in writing systems as some studies reported previously (Muscalu and Smiley, 2018; Iniesta et al., 2021). Particularly, this research did not use identical cognates. Instead, the target cognates slightly differ from the participants’ L1 Chinese or L2 English to guarantee the learning process. In this way, our data add new evidence to both the dissociable facilitative role of the L1 and the L2 in recognition and production.

Nevertheless, different from some studies (Muscalu and Smiley, 2018; Iniesta et al., 2021), the cognate interference is not found either with the L1-L3 or L2-L3 cognates in this study. Both L1 and L2 experience has been found to facilitate novel word learning. A possible reason is that the new words were taught and tested on the same day instead of a prolonged period. There was no sleep time for the participants, during which the lexical consolidation and competition would happen (Lindsay and Gaskell, 2013). The current study can be regarded as further evidence of the short-term facilitation advantage of bilingual experience (Marecka et al., 2021). The more similarities shared by the word to-be-learned and the words learned, the easier it can be learned. In the interview immediately after the experiment, all the participants reported that they had tried involuntarily to refer to the L1 or L2 original word of the cognates, especially during the learning phase. Additionally, participants reported more analytic strategies in learning L1-L3 cognates rather than the L2-L3 cognates. Therefore, learning cognates benefits from form overlap and form meaning overlap of the previously learned words. Learners were able to utilize the overlap in form and form-meaning mapping. The bilingual experience facilitates learning both L1-L3 cognates and L2-L3 cognates. In this way, L3 word learning may not be parasitic in a certain language. But rather, it is a process of building new lexical knots with language experience, even though the bilinguals acquire their L2 mainly in classroom contexts (Hirosh and Degani, 2018).

### The facilitation-and-interference effect of bilingual experience In learning false cognates

Learning false cognates were expected to entail competing processes in the direct effects of bilingual experience (Fang and Perfetti, 2017; Elias and Degani, 2022). Learning false cognates may benefit from the form overlap, but also need to overcome the meaning interference of words in the acquired language. Since L1 and L2 However, the role of bilingual experience could have been mixed with cross-linguistic similarity and language complexity. To disentangle the confusion of cross-linguistic similarity, this study has researched into the bilingual experience of orthographic difference, i.e., the logographic and alphabetic words. Through such an approach, the direct effects from L1 and L2 can be individually traced. Moreover, the confusing influence of the complexity of the logographic and alphabetic writing systems has been excluded by using the logographic and alphabetic non-cognates as baselines, respectively. Therefore, the facilitation and interference effects have been examined with the same writing system in a within-subject way. The current results of learning false cognates reveal quite different direct effects of L1 and L2 experience on novel word learning. As for the L1-L3 false cognates, the facilitation from L1 form overlapping overcomes the interference from L1 form-meaning mismatch. In both the recognition and production tasks, L1-L3 false cognates were learned significantly better than the non-cognates. However, concerning the L2-L3 false cognates, their learning outcome is almost the same as the non-cognates. Thus, the facilitation and interference from L2 are close to an equal balance. Taken together, there seems to be stronger facilitation from L1 experience rather than L2 experience when bilinguals are learning the mismatched novel words, i.e., the false cognates. The L1 facilitation outperforms its interference with a possibly better and more accurate inhibition instead of higher inhibition of the logographic form-meaning mappings from the prior knowledge (Mulík and Carrasco-Ortiz, 2021).

These results are partially similar to the research of Marecka et al. (2021), in which the learning of false cognates benefits from the overlap in L1-L2 form and is not harmed by L1 interference. In the current research, we found that novel word learning may have weaker facilitation from L2 form overlapping or stronger interference from L2 form-meaning mismatch. When comparing the recognition and production tasks of learning L2-L3 cognates, the production task shows a slightly better learning outcome. Such an advantage in production task over recognition task suggests the potential of a stronger interference from L2 form-meaning mismatch rather than a weaker facilitation from L2 form overlapping. In another word, the semantic discrepancy leads to more difficulty for L2 similar words. However, there is a significant facilitation from L1 experience in learning L1-L3 false cognates. In the production tasks, the learning of false cognates shows a very significant advantage over the learning of non-cognates, while in the production task, such an advantage just reaches the level of being significant. Therefore, the L1-L3 form-meaning mismatch also exerts an interferent effect on novel word learning, but it seems to be much weaker than the L1 facilitation. Similar L1 facilitation in learning false cognates has also been reported in learning novel words both as an L2 and an L3 (Hirosh and Degani, 2021; Marecka et al., 2021). In the study of Hirosh and Degani (2021), they found that learning false cognates through L1 translations was superior to learning them through L2 translations. Taken together, the direct effects from L1 experience seem to exert more facilitation than interference when learning the L1 form-meaning mismatch, while learning the L2 form-meaning mismatch seems to suffer more from its interference effect. Notably, our study employed paired–associate learning with pictures. In both the learning and testing phases, the facilitation and interference effects are not triggered by explicit translations. A possible reason for such learning outcomes may be that the L1 and L2 bilingual experience is quite different concerning their learning conditions, automaticity levels, etc. Therefore, the L2 form-meaning mapping is weaker than the L1 form-meaning mapping. In the interview, participants also reported that among the three distractors in the recognition task, they were rarely confused by the phonetic distractors, but they were mostly misled by the semantic distractors with the graphic distractors as the second most misleading ones. In sum, the current study suggests that L1 and L2 play quite different roles as the direct effects of bilingual experience. The better facilitation effects from L1 may derive from a better inhibition rather than higher inhibition. These findings add new evidence to the facilitation-and-interference mechanism of bilingual experience.

## Conclusion

### Findings of the study

To the best of our knowledge, the present study is the first to systematically disentangle the influence of bilingual experience *via* examining L1-L3 and L2-L3 cognate status within the same bilingual participants. The L1 and L2 experience has been analyzed, respectively, through comparing the cognates and the false cognates with the baseline of non-cognates within the same writing system to avoid mingling with the different complexity of logographic and alphabetic words. Our results show that the dominant L1 and non-dominant L2 can exert dissociable direct effects as facilitation for learning the form-meaning closely matched novel words, i.e., the cognates. However, in our research, learning the form-meaning mismatch, i.e., the false cognates, reveals the sophistication of the facilitation-and-interference effects sourced from bilingual experience. The form-meaning mismatch potentially triggers interference from both L1 and L2. But the interference is compensated by the facilitation from L1 and L2 prior knowledge and the form-meaning mapping skills. It’s worth noticing that the current study provides new evidence to the different subtlety of inhibition with a more accurate inhibition of L1 form-meaning mismatch and a less accurate inhibition of L2 form-meaning mismatch, thus resulting in the different degrees of facilitation-and-interference effect from bilingual experience. These findings carry potential educational implications in that learning novel words depends on substantial bilingual experience and requires a fuller understanding of the subtle difference in the facilitation and interference from L1 and L2. Such findings may provide some insights into foreign language teaching in different contexts (Cenoz et al., 2021; Chen et al., 2022).

### Limitations and future study

Firstly, the study is limited to the learning process of paired–associates learning without teacher instruction. Further teaching experiment is needed to identify the cost and benefit of teaching logographic and alphabetic novel words through the dominant and non-dominant languages in different teaching contexts. In addition, according to Marecka et al. (2021), the nonwords are all concrete nouns due to the limitation of the meaning represented by pictures. Therefore, adding abstract nouns, verbs, adjectives, and other types of words would increase the ecological validity of the present findings. Thirdly, we created alphabetic and logographic nonwords based on English and Chinese. However, there are still more writing systems that deserve our further attention. A power analysis can be added to decide the number of participants to address more complicated language experience, such as trilingual experience.

## Data availability statement

The original contributions presented in the study are included in the article/Supplementary material, further inquiries can be directed to the corresponding author.

## Ethics statement

The studies involving human participants were reviewed and approved by School of Foreign Languages, South China University of Technology. The patients/participants provided their written informed consent to participate in this study.

## Author contributions

YC conceived, designed the study, managed and coordinated responsibility for the research activity planning and execution, provided financial support, and drafted the manuscript. HX prepared the stimuli, recruited the participants, implemented the experiment, and performed the statistical analysis. RD and WZ recruited the participants, implemented the experiment, and collected data. YC and HX interpreted the data, wrote the article, and approved the submitted version. All authors contributed to the article and approved the submitted version.

## Funding

This work was partly supported by the Department of Education of Guangdong Province (CN; project number 2020GXJK375) and the National Planning Office of Philosophy and Social Science (project number 21BYY052).

## Conflict of interest

The authors declare that the research was conducted in the absence of any commercial or financial relationships that could be construed as a potential conflict of interest.

## Publisher’s note

All claims expressed in this article are solely those of the authors and do not necessarily represent those of their affiliated organizations, or those of the publisher, the editors and the reviewers. Any product that may be evaluated in this article, or claim that may be made by its manufacturer, is not guaranteed or endorsed by the publisher.

## Footnotes

^1^ https://www.blcu.edu.cn

^2^ https://dictionary.cambridge.org/us/dictionary/english/

^3^ https://cn.bing.com/images/

^4^ https://osf.io/kzsd5/
