# Supplementary figures and images for: How does bilingual experience influence novel word learning? Evidence from comparing L1-L3 and L2-L3 cognate status
Source: Front Psychol. 2022 Nov 24;13:1003199. doi: 10.3389/fpsyg.2022.1003199 (PMC9731340; doi:10.3389/fpsyg.2022.1003199)

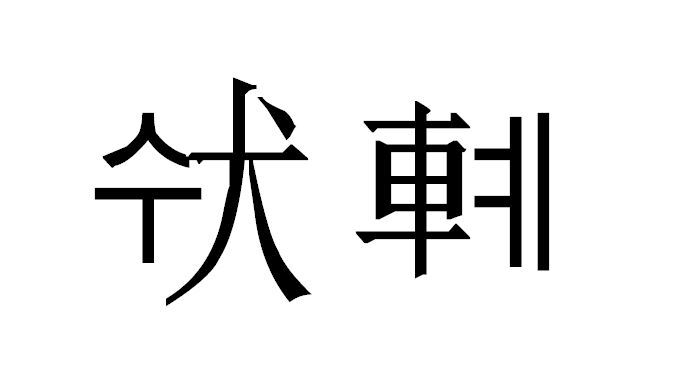

Supplement: Supplementary file 2 [file Data_Sheet_2.ZIP › logographic nonwords (picture)/卡车.jpg]

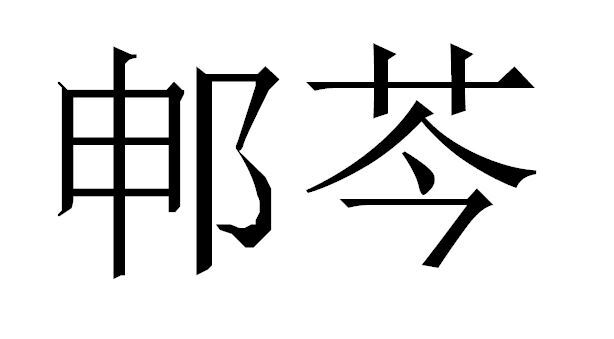

Supplement: Supplementary file 2 [file Data_Sheet_2.ZIP › logographic nonwords (picture)/呻吟.jpg]

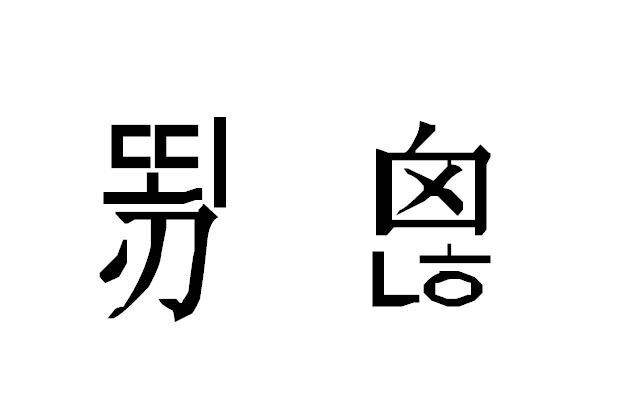

Supplement: Supplementary file 2 [file Data_Sheet_2.ZIP › logographic nonwords (picture)/天鹅.jpg]

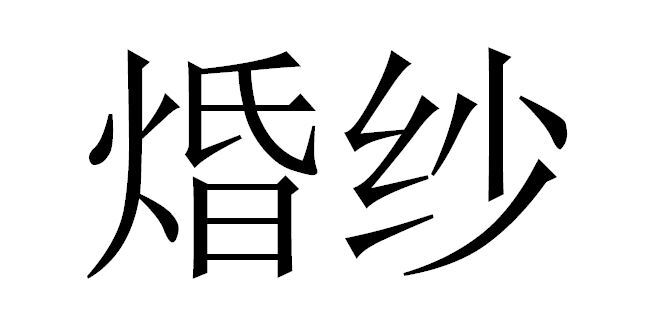

Supplement: Supplementary file 2 [file Data_Sheet_2.ZIP › logographic nonwords (picture)/婚纱.jpg]

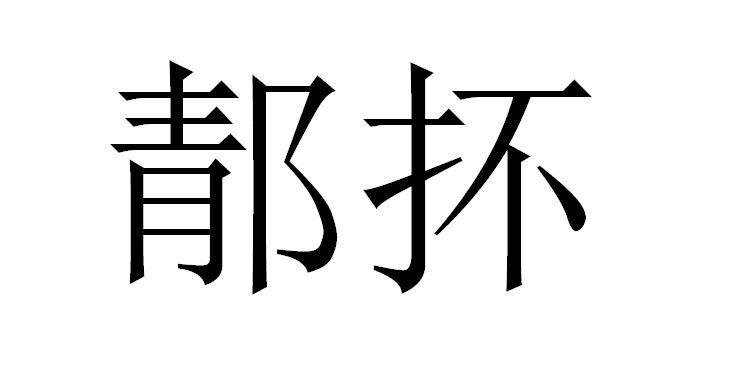

Supplement: Supplementary file 2 [file Data_Sheet_2.ZIP › logographic nonwords (picture)/情怀.jpg]

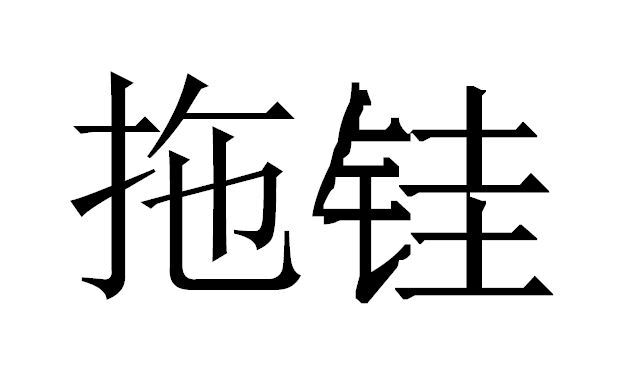

Supplement: Supplementary file 2 [file Data_Sheet_2.ZIP › logographic nonwords (picture)/拖鞋.jpg]

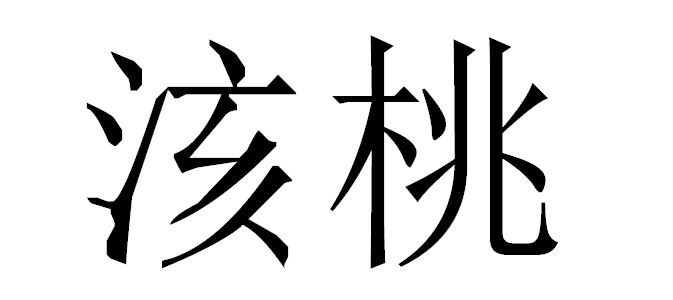

Supplement: Supplementary file 2 [file Data_Sheet_2.ZIP › logographic nonwords (picture)/核桃.jpg]

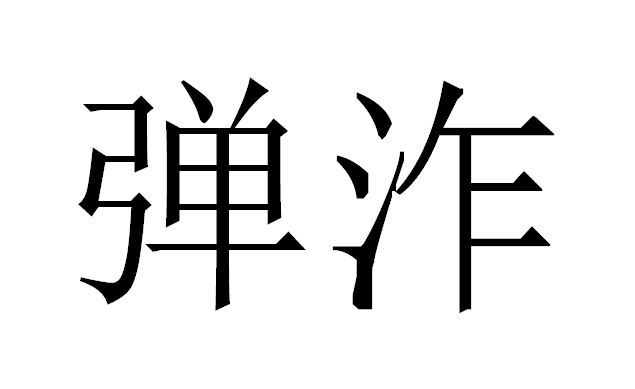

Supplement: Supplementary file 2 [file Data_Sheet_2.ZIP › logographic nonwords (picture)/炸弹.jpg]

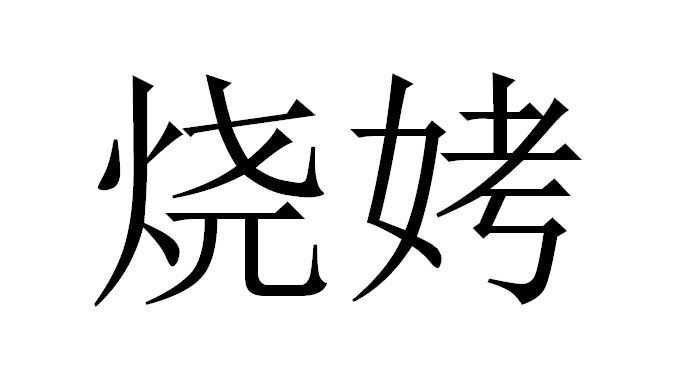

Supplement: Supplementary file 2 [file Data_Sheet_2.ZIP › logographic nonwords (picture)/烧烤.jpg]

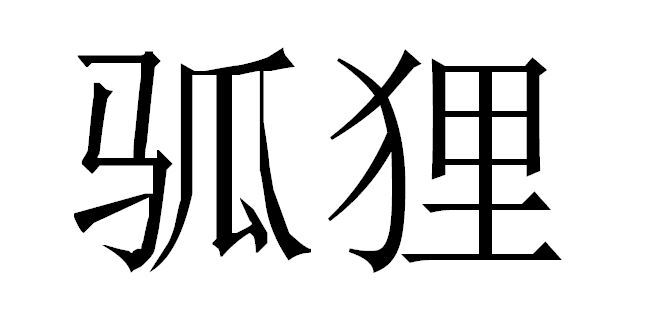

Supplement: Supplementary file 2 [file Data_Sheet_2.ZIP › logographic nonwords (picture)/狐狸.jpg]

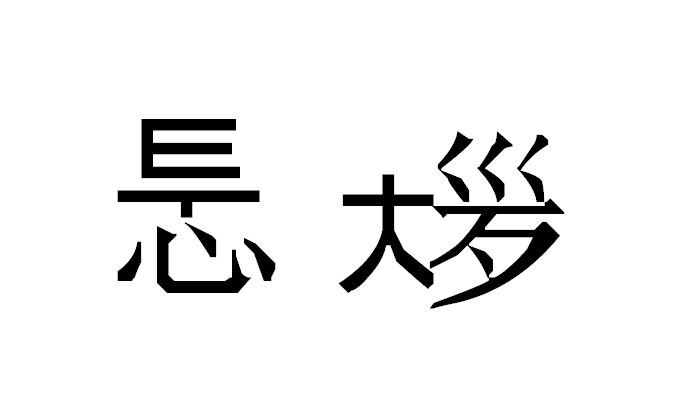

Supplement: Supplementary file 2 [file Data_Sheet_2.ZIP › logographic nonwords (picture)/电线.jpg]

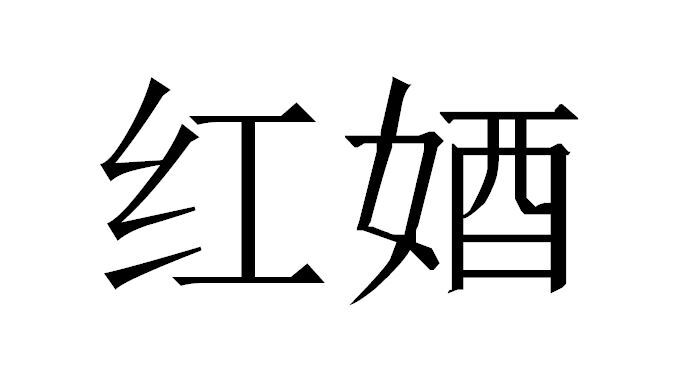

Supplement: Supplementary file 2 [file Data_Sheet_2.ZIP › logographic nonwords (picture)/红酒.jpg]

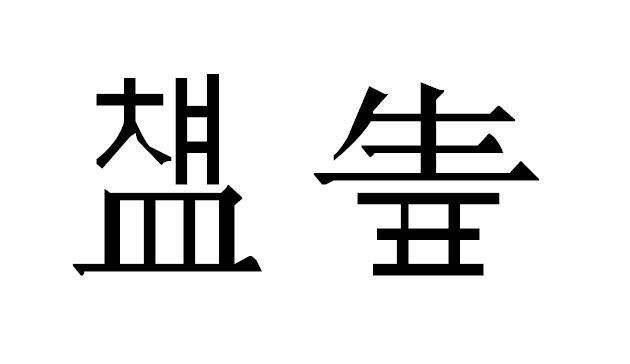

Supplement: Supplementary file 2 [file Data_Sheet_2.ZIP › logographic nonwords (picture)/腰带.jpg]

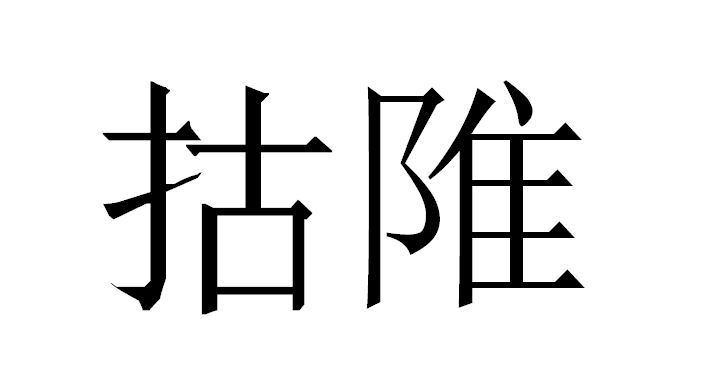

Supplement: Supplementary file 2 [file Data_Sheet_2.ZIP › logographic nonwords (picture)/苦难.jpg]

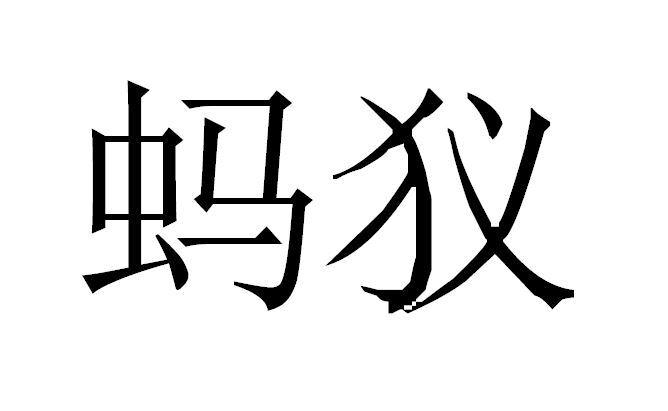

Supplement: Supplementary file 2 [file Data_Sheet_2.ZIP › logographic nonwords (picture)/蚂蚁.jpg]

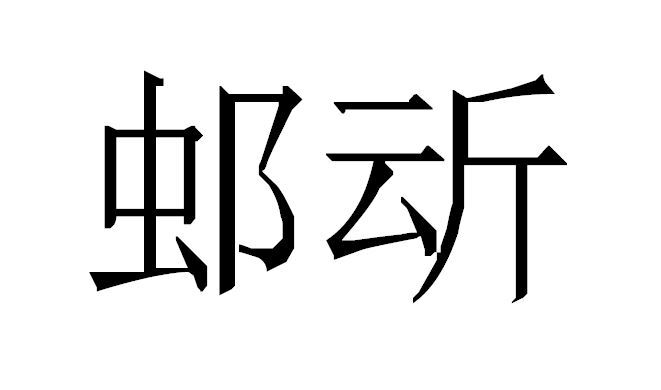

Supplement: Supplementary file 2 [file Data_Sheet_2.ZIP › logographic nonwords (picture)/触动.jpg]

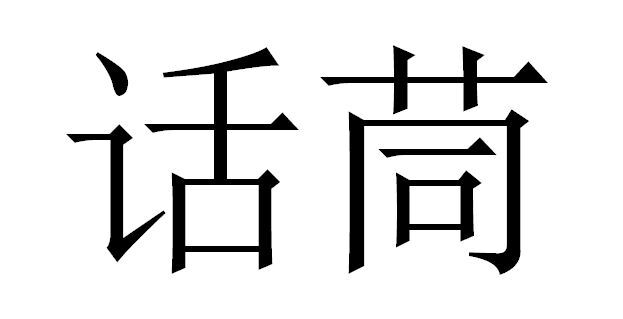

Supplement: Supplementary file 2 [file Data_Sheet_2.ZIP › logographic nonwords (picture)/话筒.jpg]

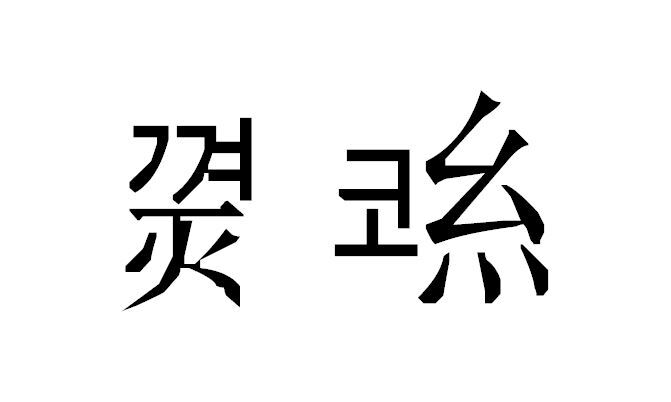

Supplement: Supplementary file 2 [file Data_Sheet_2.ZIP › logographic nonwords (picture)/轮胎.jpg]

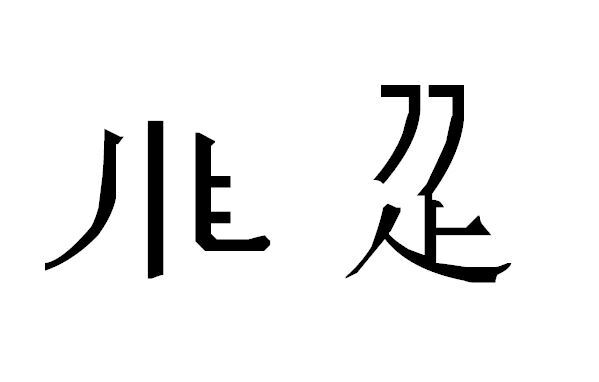

Supplement: Supplementary file 2 [file Data_Sheet_2.ZIP › logographic nonwords (picture)/邮票.jpg]

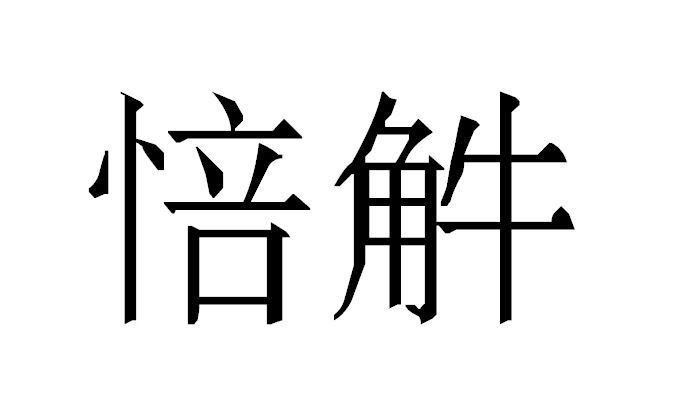

Supplement: Supplementary file 2 [file Data_Sheet_2.ZIP › logographic nonwords (picture)/部件.jpg]

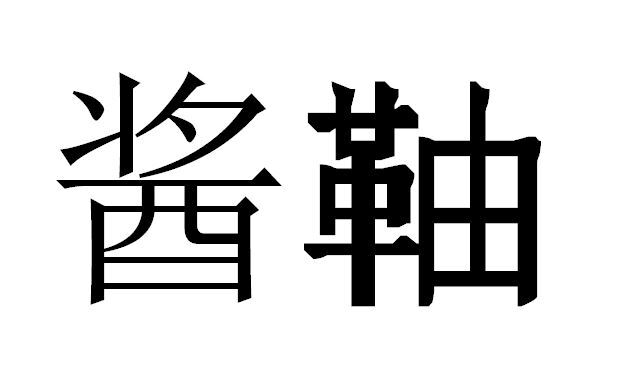

Supplement: Supplementary file 2 [file Data_Sheet_2.ZIP › logographic nonwords (picture)/酱油.jpg]

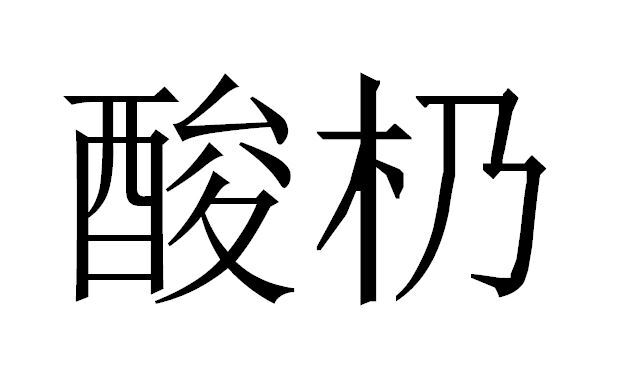

Supplement: Supplementary file 2 [file Data_Sheet_2.ZIP › logographic nonwords (picture)/酸奶.jpg]

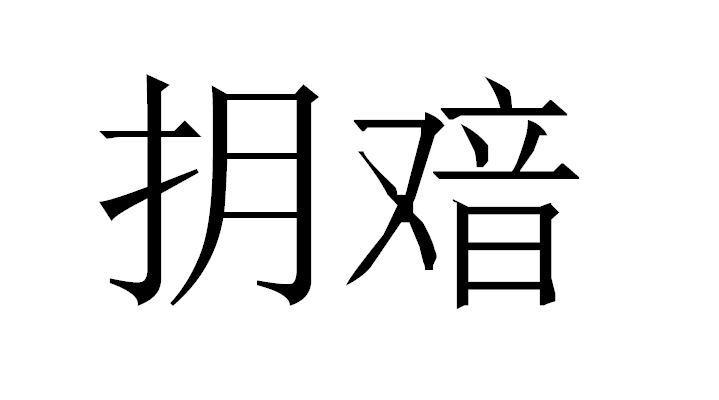

Supplement: Supplementary file 2 [file Data_Sheet_2.ZIP › logographic nonwords (picture)/阴暗.jpg]

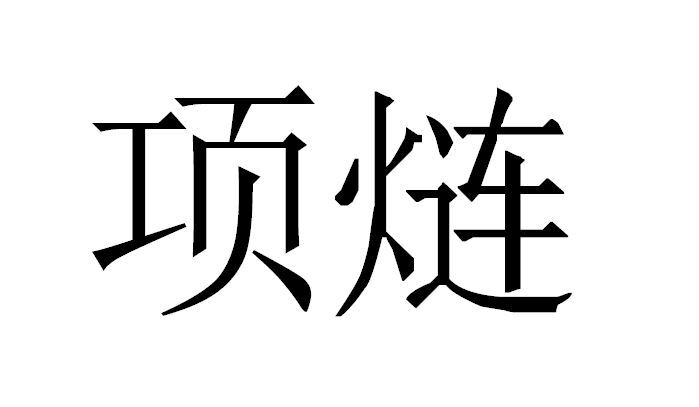

Supplement: Supplementary file 2 [file Data_Sheet_2.ZIP › logographic nonwords (picture)/项链.jpg]
